# Complement Activation-Related Pathophysiological Changes in Anesthetized Rats: Activator-Dependent Variations of Symptoms and Mediators of Pseudoallergy

**DOI:** 10.3390/molecules24183283

**Published:** 2019-09-09

**Authors:** László Dézsi, Tamás Mészáros, Erik Őrfi, Tamás G. Fülöp, Mark Hennies, László Rosivall, Péter Hamar, János Szebeni, Gábor Szénási

**Affiliations:** 1 Nanomedicine Research and Education Center, Institute of Translational Medicine, Semmelweis University, 1089 Budapest, Hungary; 2 SeroScience LCC., 1089 Budapest, Hungary; 3 TECO Development GmbH, 53359 Rheinbach, Germany; 4 Institute of Translational Medicine, Semmelweis University, 1089 Budapest, Hungary; 5 Nephrology Center, Institute of Translational Medicine, Semmelweis University, 1089 Budapest, Hungary; 6 Institute of Translational Medicine, Semmelweis University, 1094 Budapest, Hungary; 7 Institute for Translational Medicine, Medical School, University of Pécs, 7624 Pécs, Hungary; 8 Department of Nanobiotechnology and Regenerative Medicine, Faculty of Health, Miskolc University, 3505 Miskolc, Hungary

**Keywords:** complement activation, infusion reactions, zymosan, cobra venom factor, amphotericin B, blood pressure, blood cell count, thromboxane

## Abstract

Complement (C) activation can underlie the infusion reactions to liposomes and other nanoparticle-based medicines, a hypersensitivity syndrome that can be partially reproduced in animal models. However, the sensitivities and manifestations substantially differ in different species, and C activation may not be the only cause of pathophysiological changes. In order to map the species variation of C-dependent and -independent pseudoallergy (CARPA/CIPA), here we used known C activators and C activator liposomes to compare their acute hemodynamic, hematological, and biochemical effects in rats. These C activators were cobra venom factor (CVF), zymosan, AmBisome (at 2 doses), its amphotericin B-free vehicle (AmBisombo), and a PEGylated cholesterol-containing liposome (PEG-2000-chol), all having different powers to activate C in rat blood. The pathophysiological endpoints measured were blood pressure, leukocyte and platelet counts, and plasma thromboxane B2, while C activation was assessed by C3 consumption using the Pan-Specific C3 assay. The results showed strong linear correlation between C activation and systemic hypotension, pointing to a causal role of C activation in the hemodynamic changes. The observed thrombocytopenia and leukopenia followed by leukocytosis also correlated with C3 conversion in case of C activators, but not necessarily with C activation by liposomes. These findings are consistent with the double hit hypothesis of hypersensitivity reactions (HSRs), inasmuch as strong C activation can fully account for all symptoms of HSRs, but in case of no-, or weak C activators, the pathophysiological response, if any, is likely to involve other activation pathways.

## 1. Introduction

One of the promising new directions in pharmacotherapy is the use of nanoparticulate vehicles for controlled, targeted drug delivery [1,2]. Beside major advances, however, one unsolved problem with these nanomedicines is that they tend to cause infusion reactions, which are acute, IgE-independent hypersensitivity reactions (HSRs) [3,4,5]. The percentage of patients suffering from HSRs varies considerably depending on the physicochemical features of the nanodrug administered and the type of side effect. In case of liposomal amphotericin B formulations, 20% of patients are estimated to produce one or fewer symptoms including i) chest pain, dyspnea, and hypoxia; ii) severe abdominal, flank, or leg pain; and iii) flushing and urticaria [6]. Complement (C) activation is one explanation for these reactions, leading to the term C activation-related pseudoallergy (CARPA) [7]. However, recent studies in pigs [8] and mice [9] raised the possibility of C-independent mechanisms as well, referred to as C-independent pseudoallergy (CIPA). At this time, we cannot exclude the possibility that the apparent absence of C activation in these cases was due to assay failure, such as low sensitivity and/or improper test and/or incorrect sampling times, and, in fact, C activation was present, just not detected. An example for the latter possibility was seen in the case of polystyrene nanoparticle-induced HSRs in pigs, wherein C activation in vitro was detected by analysis of particle opsonization [10], but not by ELISA of C split products [8]. Accordingly, to remain open for both possibilities, our current hypothesis (“double hit theory”) is that CARPA and CIPA may proceed in parallel, and their relative contributions may differ in different species [9] and under different experimental conditions [4,10].

Previous studies on liposome-induced CARPA in rats used liposome-encapsulated hemoglobin (LEH) [11,12,13,14,15], liposomes containing 71% cholesterol (HC-MLV) [16], amphotericin B-containing liposomes (AmBisome), and a polyethylene glycol (PEG)-grafted liposome preparation wherein the 2 kDa-PEG was anchored to the membrane via cholesterol (PEG-2000-chol) [17,18], all these vesicles having different physicochemical properties and different powers to activate C in rat blood. As a positive control, the well-known C activator yeast membrane extract, zymosan, was applied. The present study extends these previous investigations on the acute pathophysiological effects of known C activator macromolecules and different C activator liposomes in rats. In particular, here we added cobra venom factor (CVF) among the tested C activators, which directly depletes C3, and applied a C3 consumption ELISA (“Pan C3”) besides the CH50 assay to measure C activation. While confirming the previous findings with zymosan, AmBisome and PEG-2000-chol [17], here we show that strong C activation by CVF, just like the effect of zymosan, correlates with the blood pressure changes, while the variable pathophysiological effects of different liposomes seem to show variable relationship with C activation.

## 2. Results

### 2.1. Complement Activation by the Different Reaction Inducers

**Figure 1:**
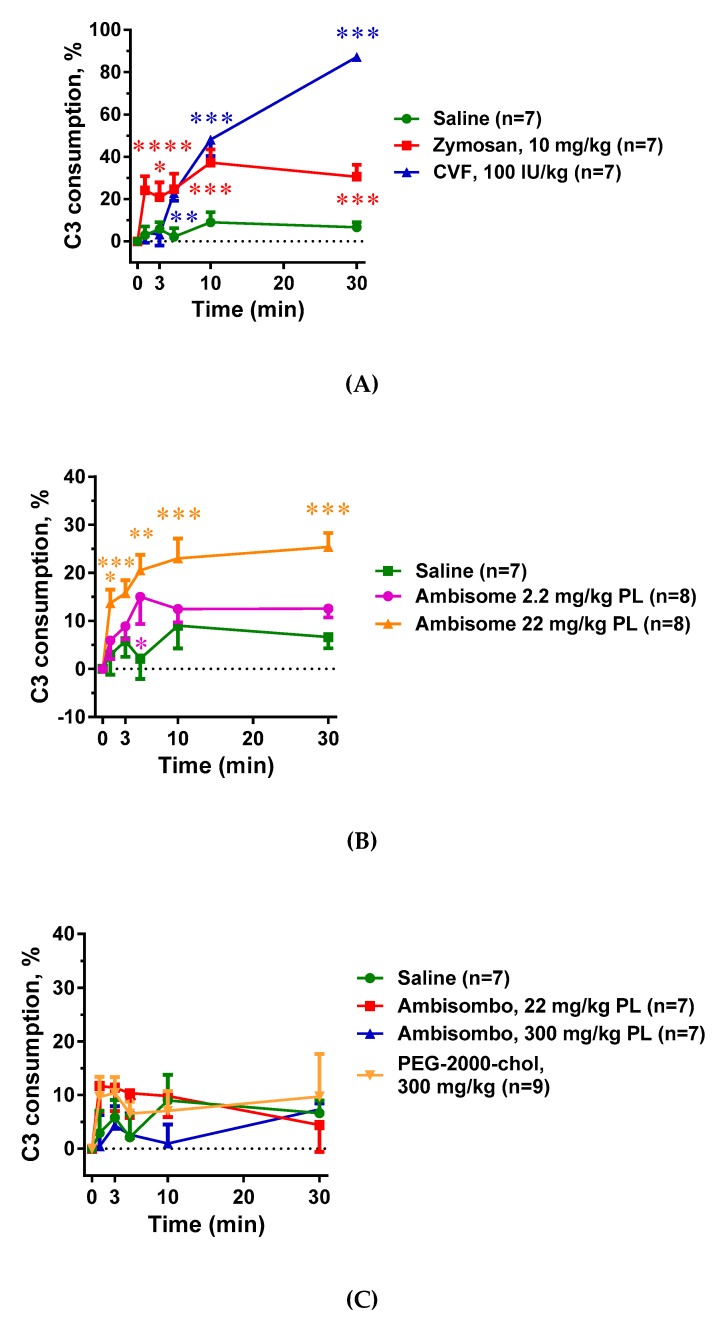
Effects of direct complement activators, amphotericin B-containing and empty liposomes, on the complement system in anesthetized rats. Complement activation was assessed as complement C3 consumption expressed as percentage decreases in C3 concentration relative to the baseline (time 0). (**A**) Effects of direct complement activators, zymosan and cobra venom factor (CVF); (**B**) Effects of the amphotericin B-containing liposome AmBisome applied at 2 different doses. (**C**) Effects of empty liposomes, AmBisombo and PEG-2000-chol. The data are mean ± SEM. Statistical analysis was performed using two-way repeated measurements ANOVA followed by Dunnett’s multiple comparisons post-hoc test. Significant differences (* = *p* < 0.05; ** = *p* < 0.01; *** = *p* < 0.001) are shown relative to the group treated with saline.

As expected, both zymosan and CVF caused massive C activation in rat blood, although the extent and kinetics of changes were different for the two activators (Figure 1A). Namely, C3 depletion reached a plateau earlier and at a lower level in the case of zymosan compared to CVF. AmBisome, at a high dose, also caused significant C activation (Figure 1B), while unexpectedly, the AmBisome-equivalent drug-free liposome AmBisombo and PEG-2000-chol liposomes did not, even at high concentrations (Figure 1C). This suggests that amphotericin B had a key role in engendering AmBisome liposomes with C activator capability, at least in rat blood in vivo, and that PEG-2000-chol, which was found to be a strong C activator in human serum [18], is not displaying this activity in rats in vivo. Overall, these data show substantial variation of in vivo C activation among the different inducers of HSR, enabling a correlation analysis with the various pathophysiological changes that these drugs and agents caused.

### 2.2. Hemodynamic Changes and Their Correlation with C Activation

**Figure 2:**
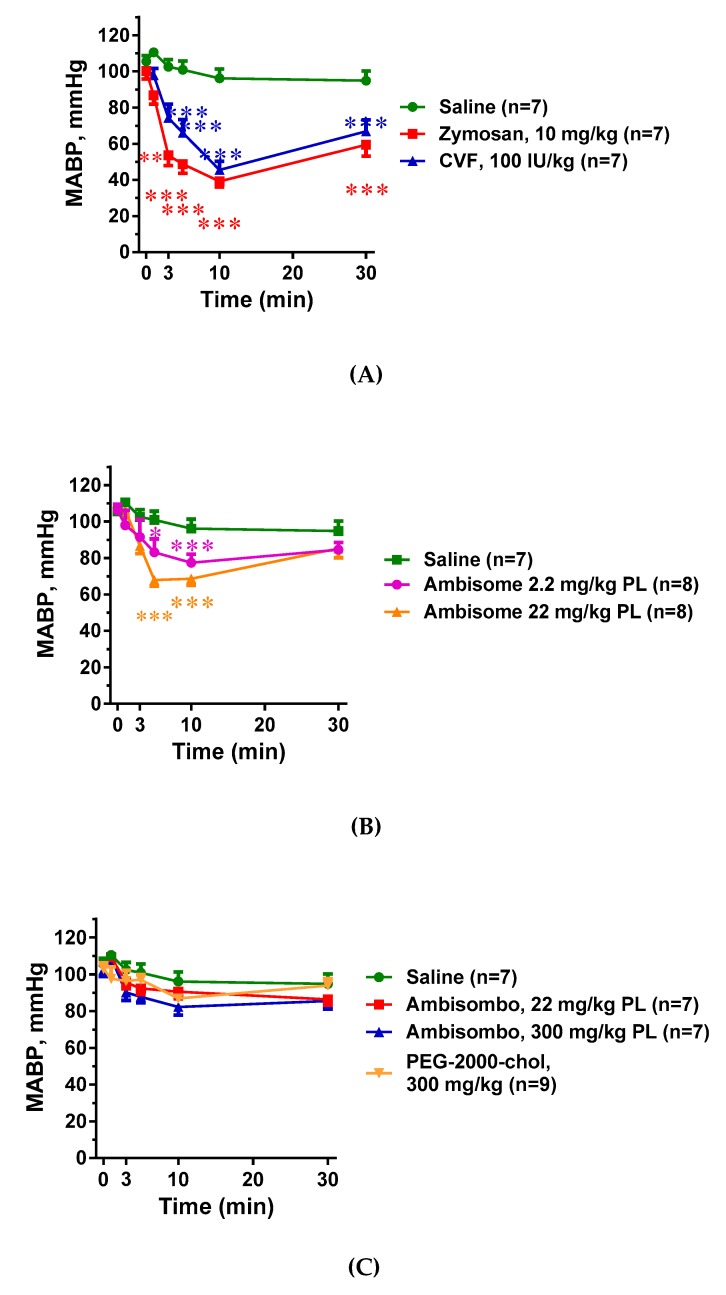

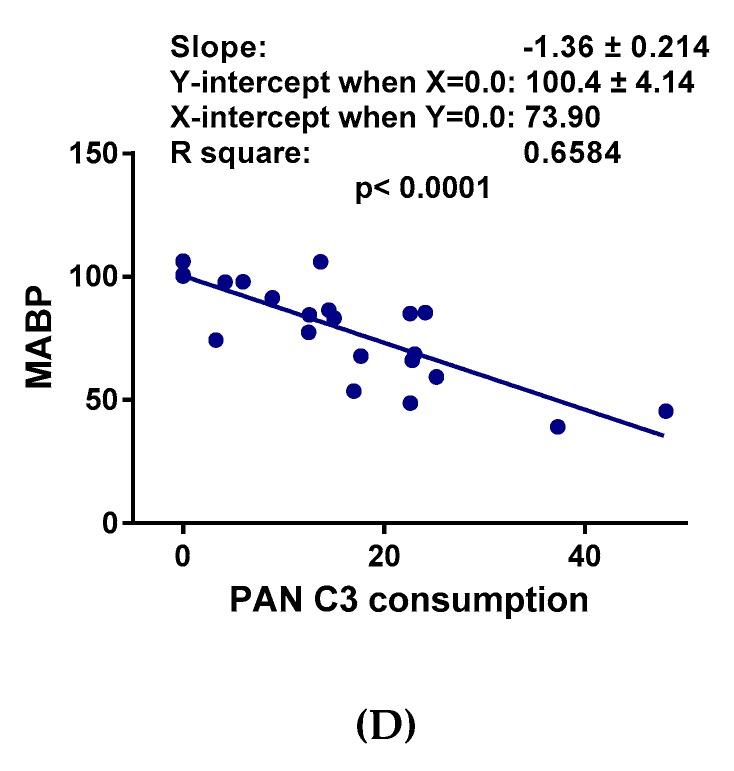
Blood pressure changes following in vivo administration of direct complement activators, amphotericin B-containing liposomes and empty vesicles. The doses and number of animals are specified in the keys; MABP, mean arterial blood pressure. (**A**) Effects of zymosan and cobra venom factor (CVF); (**B**) AmBisome at two doses; and (**C**) empty liposomes (AmBisombo at 2 doses and PEG-2000-chol). (**D**) Correlation between the lowest MABP and C3 consumption at the same time, including the data with CVF, zymosan, and AmBisome at both doses. Except for saline, CVF, and AmBisombo, the data in [17] were replotted with permission of the publisher. The data show percentages of change relative to baseline (t = 0 min), mean ± SEM. Statistical analysis was performed using two-way repeated measurements ANOVA followed by Dunnett’s multiple comparisons post-hoc test. Significant differences (* = *p* < 0.05; ** = *p* < 0.01; *** = *p* < 0.001) are shown relative to the group treated with saline.

As shown in Figure 2A, the two known C activators, zymosan and CVF, caused a major (>60%) drop of the mean arterial blood pressure (MABP) within 10 min, followed by partial recovery until the end of the 30 min observation period. Liposomal amphotericin B (AmBisome) also decreased the MABP in a dose-dependent manner (Figure 2B), but the effect was less expressed compared to the C activators. In contrast, large doses of AmBisombo and PEGylated cholesterol containing small unilamellar vesicles, PEG-2000-chol, caused only a small, statistically insignificant trend for hypotension, especially AmBisombo at 300 mg/kg (Figure 2C). Importantly, plotting the lowest MABP values against C3 consumption in the animals treated with zymosan, CVF, and AmBisome (both doses), showed highly significant linear correlation (Figure 2D), suggesting that C activation, whenever present, played a causal role in the transient hypotension.

### 2.3. Blood Cell Changes and Their Correlation with C Activation

**Figure 3:**
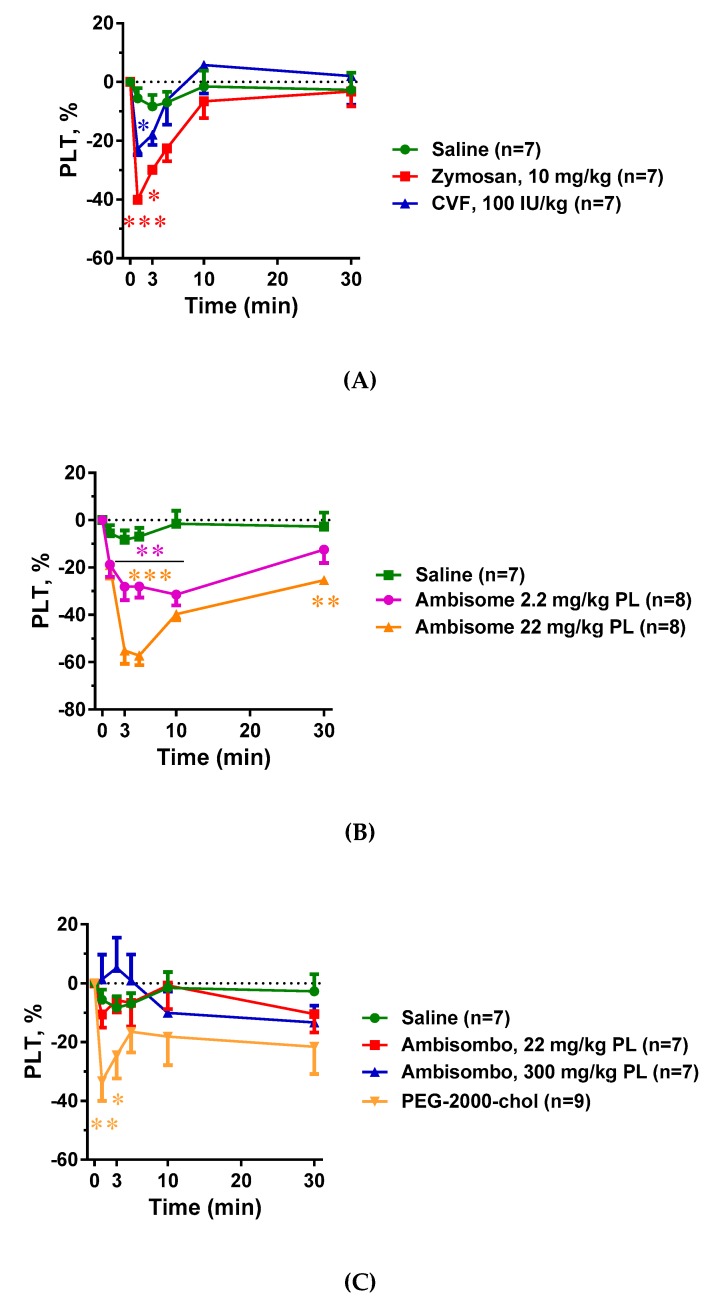
Effects of direct complement activators (**A**), amphotericin B-containing (**B**), and empty liposomes (**C**) on plasma platelet (PLT) counts in anesthetized rats. The doses and number of animals are specified in the keys. The data show percentages of change relative to baseline (t = 0 min), mean ± SEM. Statistical analysis was performed using two-way repeated measurements ANOVA followed by Dunnett’s multiple comparisons post-hoc test. Significant differences (* = *p* < 0.05; ** = *p* < 0.01; *** = *p* < 0.001) are shown relative to the group treated with saline.

**Figure 4:**
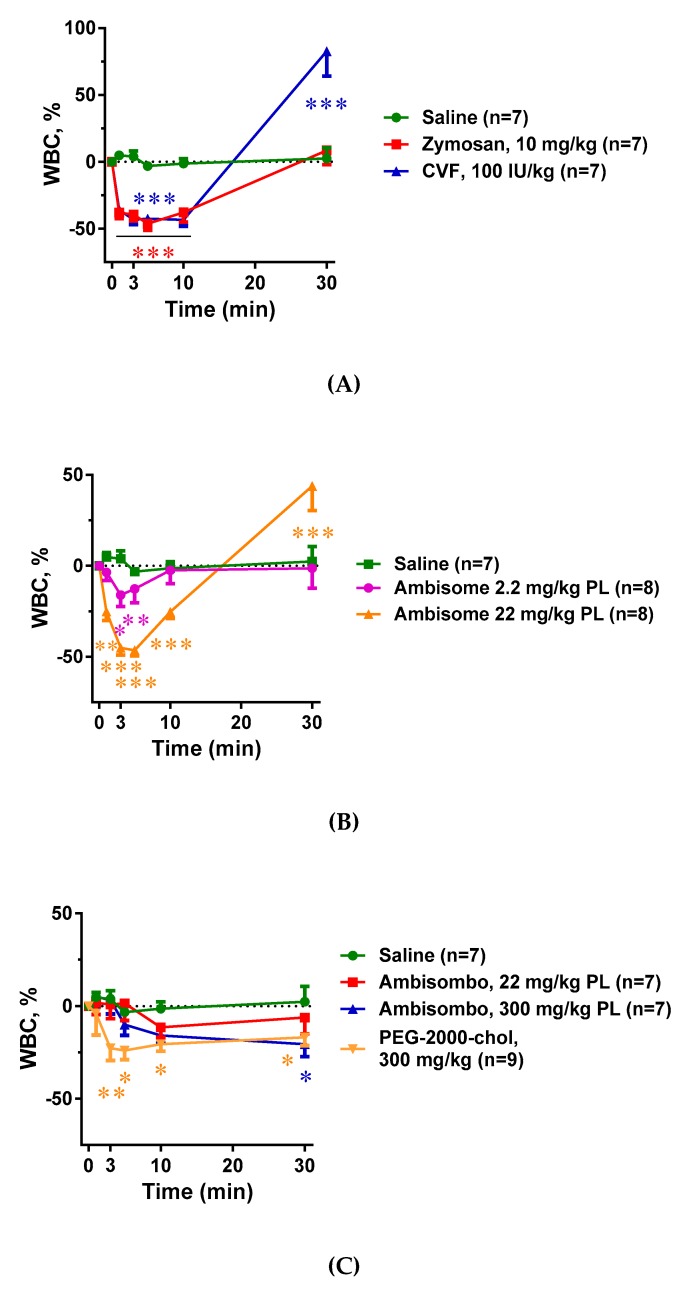
Effects of direct complement activators (**A**), amphotericin B-containing (**B**), and empty liposomes (**C**) on plasma white blood cell (WBC) counts in anesthetized rats. The doses and number of animals are specified in the keys. The data show percentages of change relative to baseline (t = 0 min), mean ± SEM. Statistical analysis was performed using two-way repeated measurements ANOVA followed by Dunnett’s multiple comparisons post-hoc test. Significant differences (* = *p* < 0.05; ** = *p* < 0.01; *** = *p* < 0.001) are shown relative to the group treated with saline.

Figure 3A–C shows that all C activator inocula, including zymosan, CVF, and AmBisome, caused significant thrombocytopenia that seemed to be proportional to C3 consumption. There was, however, one exception: PEG-2000-chol, which caused no C activation, yet it led to major thrombocytopenia comparable to those caused by CVF and high dose AmBisome (Figure 3C). The same applied to the leukopenia with compensatory leukocytosis in case of CVF and high dose of AmBisome, which was greater when C activation was larger in cases of zymosan, CVF, and AmBisome, and was present to a lesser but significant extent in cases of empty liposome activators AmBisombo and PEG-2000-chol (Figure 4A–C). These data suggest that the blood cell changes observed in this model have C-dependent as well as C-independent mechanisms of action, i.e., they may be manifestations of simultaneous CARPA and CIPA. In cases of the strong C activator zymosan, CVF, and AmBisome at large dose, CARPA may fully account for these changes, while in cases of non-C-activators AmBisombo and PEG-2000-chol, the reaction may reflect CIPA.

### 2.4. Plasma TXB2 Changes and Their Correlation with C Activation and Systemic Hypotension

**Figure 5:**
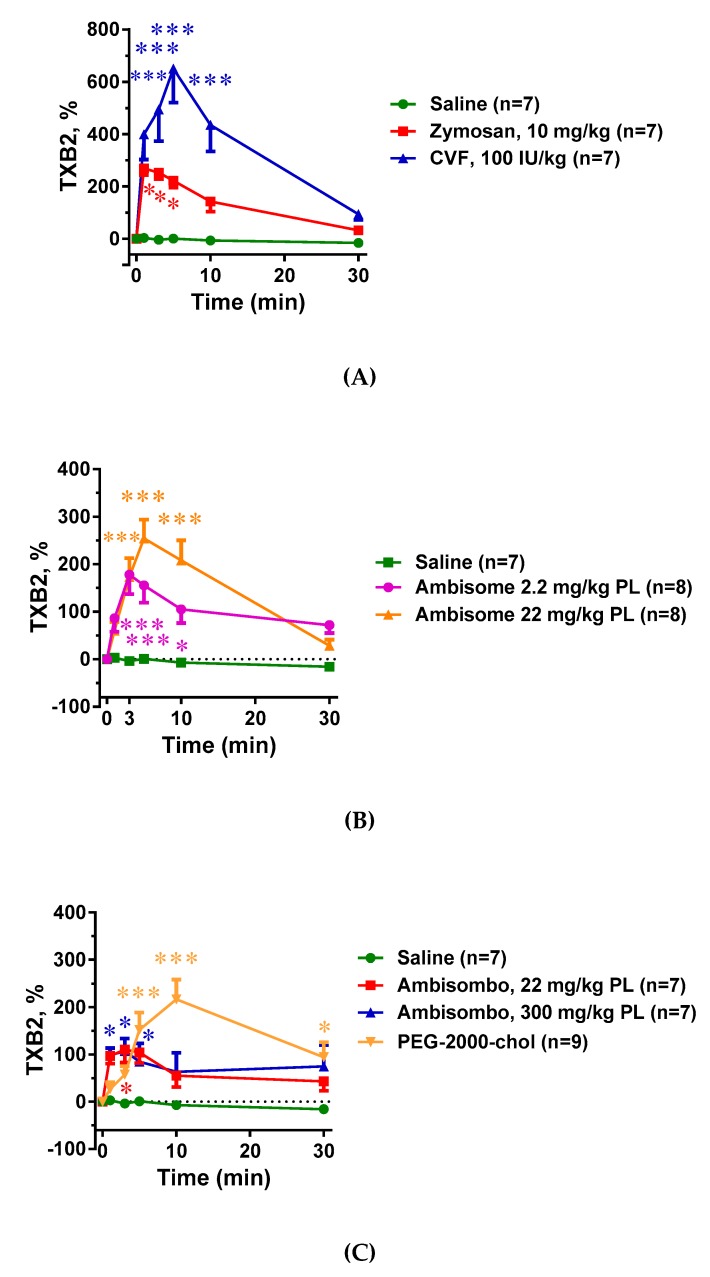
Effects of direct complement activators (**A**), amphotericin B-containing (**B**), and empty liposomes (**C**) on plasma white blood cell (WBC) counts in anesthetized rats. The doses and number of animals are specified in the keys. The data show % change relative to baseline (t = 0 min), mean ± SEM. Statistical analysis was performed using two-way repeated measurements ANOVA followed by Dunnett’s multiple comparisons post-hoc test. Significant differences (* = *p* < 0.05; ** = *p* < 0.01; *** = *p* < 0.001) are shown relative to the group treated with saline.

Just as the blood cell changes (Figure 3 and Figure 4), all C activator inocula, including zymosan, CVF, and AmBisome, caused significant elevation of plasma TXB2, and the greater the C3 consumption, the larger the change in TXB2 concentration. (Figure 5A–C) However, non-C-activator liposomes also caused significant rises in TXB2, particularly PEG-2000-chol (Figure 5C), which points to C-independent triggering of TXA2 release.

## 3. Discussion

This study shows that in vivo administration of different C activator macromolecules and liposomes caused qualitatively similar, but quantitatively different hypersensitivity symptoms, including transient hypotension, increases in plasma TXB2 concentration, thrombocytopenia, and leukopenia that was followed by compensatory leukocytosis in cases of CVF and a high dose of AmBisome. Strong C activation implying 20–40% C3 consumption within 10 min (caused by CVF, zymosan, and high-dose AmBisome) showed clear association with all the above symptoms, suggesting that C activation plays a critical role in the downstream events of HSRs over a certain intensity threshold. In cases of mild C activator or C-stealth liposomes (AmBisombo, PEG-2000-chol), which entailed <15% C3 consumption in 10 min, the C-dependence of different side effects varied with different liposomes and symptoms. Notably, despite the lack of major C activation and hypotensive effect, AmBisombo and PEG-2000-chol liposomes caused major leukopenia and TXB2 release, and PEG-2000-chol liposomes also caused significant thrombocytopenia. These divergent effects suggest concurrent involvement of C-dependent and C-independent processes in HSRs, referred to as CARPA and CIPA [4,9].

Regarding the possibility of pure CIPA, however, it needs to be emphasized that the absence of C3 consumption in the PAN-C3 assay does not exclude C activation; it just indicates the absence of strong C activation which visibly depletes C3. This is because C3 has a very high plasma level whose reduction requires massive C activation. Thus, the assay does not necessarily detect low levels of anaphylatoxin production, which has biological effects without changing plasma C3. At this stage, we cannot rule out a small, but critical contribution of CARPA to HSRs seemingly representing mainly CIPA. This proposal is consistent with the finding that AmBisombo and AmBisome induced similar rises in TXB2, although only the C activator AmBisome caused significant hypotension, which suggests synergism between C activation and C-independent TXA2 action. That C activation has hemodynamic effects in rats independent of thromboxane release is known from other studies, e.g., the C3a receptor agonist induced hypertension or the C5a receptor agonists induced hypotensive effect [19]. In addition, the hypotensive response of zymosan was largely prevented by a platelet activating factor (PAF) antagonist [20], which implies a major role of PAF, which can also be induced by C5a. Based on our results and previous reports, we propose therefore that C activation contributes to hypotension via several different pathways that involve different membrane receptors. The same applies to the blood cell changes, as it has been shown that stimulation of C3aR increases, while C5aR decreases white blood cell counts in rats [19,21].

As for the C dependence of TXB2 release, the suppression of plasma TXB2 by the C inhibitor soluble complement receptor 1 and by depletion of C by pretreatment with CVF [15] attest to a causal relationship, while our finding in the present study on TXB2 rise by PEG2000-Chol appears C independent. Likewise, pretreatment with CVF prevented the LEH-induced thrombocytopenia and sequestration of platelets in the lungs and liver [13], while in the present study, PEG2000-Chol had a thrombocytopenic effect without major C activation.

Taken together, our results are consistent with a causal role of C activation in the HSRs caused by C activator drugs and agents in individuals sensitive for C activation, but refines this concept with the constraint that the extent of activation is critical, and only extensive C activation can be considered as major or sole cause of HSRs. A similar conclusion was drawn in a clinical trial with liposomal doxorubicin (Doxil), which showed significant correlation between severe HSRs and C activation only in patients displaying extensive formation of terminal C complex, an end-product of C activation [22]. Likewise, most recently we have shown significant correlation between C activation by Doxil and pseudo-anaphylaxis in pigs when the animals were sensitized for C activation by increasing the anti-PEG antibody level in their blood by way of immunization [23]. These findings fit the double “hit” hypothesis of HSRs, which claims that the broad spectrum of HSR symptoms reflects the combined effects of C-dependent and C-independent stimulation in a variety of allergy-mediating cells. The relative contributions of CARPA and CIPA may vary for different drugs and animal models, and also depend on the experimental conditions [4,23].

This study has the limitation that we measured C3 consumption indicating global complement activation, rather than measuring anaphylatoxin (C3a, C5a) levels, opsonic scission products (C3b and derivatives), activation pathway markers (Bb, C4d), or other C activation by-products that may have an impact on HSRs [23]. We assayed plasma C3a concentration using a low sensitivity ELISA kit, which failed to detect plasma C3a concentration and a high sensitivity ELISA kit, the results of which were not reproducible (data not shown). For the time being, the lack or unsuccessful use of commercial rat-specific ELISAs for these analytes represent a technical barrier to achieving these goals. Likewise, we did not study the mechanism of C-independent changes in light of the great number of potential pathways [8,23]. Furthermore, the finding in the present in vivo rat study of no significant C activation by PEG-2000-chol liposomes contradicts our previous report where these liposomes were strong C activators in human serum in vitro [18]. Such a discrepancy in the findings is likely due to species differences. Further experiments addressing these issues and questions will hopefully shed more light on the molecular details of CARPA/CIPA duality in HSRs.

## 4. Materials and Methods

### 4.1. Animals

Male Wistar rats weighing 270–350 g were purchased from Toxicoop Ltd. (Budapest, Hungary). The animals were kept in a temperature- and humidity-controlled room with free access to standard rodent chow (Altromin standard rodent diet, GmbH & Co. KG, Lage, Germany) and tap water. The animal cages were kept in a room maintained on a 12 h/12 h light/dark cycle with lights on at 6:00 a.m., and the temperature and relative humidity were controlled at 21–24 °C and 40–60%, respectively. A minimum of one-week adaptation was allowed upon arrival before starting the experiments.

### 4.2. Ethical Approval

All procedures were performed in accordance with guidelines set by the National Institutes of Health (USA) and the Hungarian law on animal care and protection. The protocol was approved by the Institutional Ethical Committee for Animal Care and Use of Semmelweis University (registration number: PEI-001/3948-6/2014).

### 4.3. Liposomes and Chemicals

PEG-2000-chol liposomes of 100 nm size and containing polyethyleneglycol and cholesterol in 15%/85% *V/V* ratio were prepared using a film extrusion method [18]. AmBisombo liposomes containing dimyristoyl phosphatidylglycerol (31.1 mg/mL), cholesterol (22.35 mg/mL), and phosphatidylcholine (91.55 mg/mL) were prepared with the extrusion method [24]. AmBisome (Gilead Sciences Int., Ltd., Cambridge, England) was obtained from a local pharmacy, cobra venom factor was obtained from Quidel Co. (San Diego, CA, USA), and zymosan was obtained from Sigma Hungary Ltd. (Budapest, Hungary).

### 4.4. Experimental Protocol

Rats were anesthetized with thiobutabarbital sodium (Inactin, Sigma Aldrich, Budapest, Hungary) at the dose of 120 mg/kg i.p. After the tracheostomy, tubing was inserted into the trachea to allow free airways. The left carotid artery was cannulated by PE20 tubing for blood sampling. The femoral artery and vein were cannulated with PE20 and PE50 tubing for measuring blood pressure and administration of test items, respectively.

Arterial blood pressure (MABP) was measured using a BPR-02 pressure transducer (Experimetria Ltd., Budapest, Hungary), an HG-01D BP amplifier (Experimetria Ltd., Budapest, Hungary), and the mean arterial blood pressure (MABP) was calculated. Heart rate (HR) was derived from the pulsatile blood pressure curve. The data were collected using a PowerLab data acquisition system (ADInstruments Ltd., Oxford, United Kingdom) and were recorded on a desktop computer using LabChart data analysis software (ADInstruments Ltd., Oxford, UK). A minimum of 10 min control period was recorded to confirm steady-state blood pressure and heart rate. The test items were slowly injected over 1 min into the femoral vein in a volume of 1 mL/kg. The control group received an equivalent volume of saline. Approximately 0.5 mL blood samples were collected prior to treatments (time 0) and at 1, 3, 5, 10, and 30 min after treatments.

### 4.5. Analytical Procedures

Blood was collected for the PAN-C3 assay in 1.5 mL Eppendorf tubes containing 15 µg hirudin (10 µL) and for the TXB2 assay we used K3EDTA tubes (MiniCollect^®^, Greiner Bio-One Hungary Kft, Budapest, Hungary) that contained indomethacin (10 µg/tube, 5 µL). Blood was centrifuged at 1500× *g* for 10 min at 4 °C to obtain plasma. Total plasma C3 concentration was measured after conversion of rat C3 to sC5b-9 using a MicroVue Pan-Specific C3 Reagent kit (Quidel, San Diego, CA, USA), and C3 consumption was calculated as percentage change relative to the time 0 sample. Thromboxane B2 (TXB2) was measured by ELISA (Cayman Chemical, Ann Arbor, MI, USA). Blood cell counts were measured using an Abacus hematological analyzer (Diatron MI Co., Budapest, Hungary).

### 4.6. Statistical Analysis

All data presented are means ± SEM. The treated groups were compared to the control group per time using two-way ANOVA for repeated measurements followed by Dunnett’s multiple comparisons test. Linear correlation between MABP and C3 consumption was calculated using the Pearson’s correlation coefficient. Statistical tests were performed using GraphPad Prism version 6 for Windows (GraphPad Software, La Jolla, CA, USA).
